# Mechanically induced experimental knee osteoarthritis benefits from anti-inflammatory and immunomodulatory properties of simvastatin via inhibition of matrix metalloproteinase-3

**DOI:** 10.1007/s10195-011-0154-y

**Published:** 2011-08-24

**Authors:** Erdem Aktas, Ertugrul Sener, Pınar Uyar Gocun

**Affiliations:** 1 Department of Orthopaedics, School of Medicine, Gazi University, Barış Sitesi, 2112. sok No: 7, Eskişehir Yolu/Ankara, Turkey; 2 Department of Pathology, School of Medicine, Gazi University, Ankara, Turkey

**Keywords:** Simvastatin, Anterior cruciate ligament transection, Knee osteoarthritis, Matrix metalloproteinase-3

## Abstract

**Background:**

We investigated the anti-inflammatory and immunomodulatory effect of simvastatin on articular cartilage via the inhibition of matrix metalloproteinase-3 (MMP-3), a matrix-degrading enzyme, in a mechanically induced experimental osteoarthritis (OA) animal model.

**Materials and methods:**

Twenty-seven albino Wistar rats were divided in three groups of equal number. Unphysiologic loading of articular cartilage was simulated by transecting anterior cruciate ligaments of the right knees of 18 rats consisting of groups 1 and 2. Nine animals in group 2 received orally administered simvastatin 20 mg/kg per day by gavage for 8 weeks. Animals in group 3 were sham operated. All animals were sacrificed at postoperative 8 weeks. Effects of simvastatin on disease progression was evaluated by documenting OA changes in cartilage specimens using Osteoarthritis Research Society International (OARSI) OA cartilage histopathology assessment system scores combined with the percentage of MMP-3 expression in chondrocytes.

**Results:**

Simvastatin treatment significantly down-regulated the percentage of MMP-3 expression in chondrocytes as assessed by immunohistochemistry methods. Suppression of this matrix-degrading enzyme by simvastatin also reduced OARSI scores, suggesting the potential for statins against OA progression.

**Conclusions:**

Following knee trauma, OA initiates at the molecular level in a short period of time. Irreversible structural changes in cartilage that require demanding treatment strategies led us to focus on effective measures to prevent OA. Statins have immunomodulatory and anti-inflammatory properties independent from their serum-cholesterol-lowering effects. One of these widely used drugs, simvastatin, showed beneficial effects on OA progression and extent by reducing cartilage degradation in our experimental setting. If these results are confirmed by human trials, simvastatin might be considered by orthopedic surgeons as a disease-modifying drug during the early inflammatory phase of posttraumatic OA.

## Introduction

Osteoarthritis (OA), characterized by progressive degradation of cartilage, is the most common joint disease for middle-aged and older people. In advanced stages of the disease, limb dysfunction due to joint pain, deformity, contracture, and muscle atrophy is a handicap in daily living. The high frequency and chronicity of OA makes it a substantial economic burden for patients and health care systems. Thus we focused our study on effective preventive measures. Currently, there is no disease-modifying treatment for OA; available medical treatment for early OA is based on symptomatic relief.

Although the pathophysiology of joint degeneration remains poorly understood, increase in mechanical stress and changes in biochemical factors within the affected joint are thought to be responsible for disease progression. This is a complex, multifactorial process involving cartilage catabolism and anabolism, as well as changes in other tissues in the joints, such as the synovium, subchondral bone, and tendons. Chondrocytes regulate cartilage metabolism. Homeostasis of extracellular matrix is driven mainly by enzymes secreted from these chondrocytes. As a consequence of mechanical and biochemical events, imbalance between synthesis and degradation of articular cartilage matrix results in clinical OA. Advanced molecular studies emphasize that OA is not only a degenerative disease, there is also an ongoing inflammatory process in its pathophysiology [1–5]. Matrix metalloproteinases (MMPs) and proinflammatory cytokines such as interleukin-1 (IL-1), IL-6, and tumor necrosis factor alpha (TNF-α) play an important role in this inflammatory process [6–8]. Several studies have provided evidence for a significant role of MMPs, particularly MMP-3, produced by chondrocytes in the development of cartilage degradation [9–11]. MMPs, a gene family of zinc-dependant proteases, are secreted from various cells, including chondrocytes, synovial-lining cells, neutrophils, and macrophages. In early studies, elevated levels of MMPs were found in osteoarthritic knee and joint cartilage of humans undergoing total joint replacement [12]. MMPs released by chondrocytes can be enhanced in conditions of mechanical or chemical stresses, destroying nearly all components of the cartilage matrix. Among the mediators of tissue injury, IL-1 and TNF-α are actively involved in the progression of cartilage damage and can stimulate MMP secretion from chondrocytes and synovial tissues [13–15].

Statins are competitive inhibitors of hydroxymethylglutaryl (HMG)-CoA reductase and are used worldwide as the most effective drug to reduce serum cholesterol levels by inhibiting the cholesterol biosynthesis pathway. HMG-CoA reductase catalyzes the conversion of HMG-CoA to mevalonate, a rate-limiting step in cholesterol biosynthesis. Statins have been shown to have anti-inflammatory effects that are unrelated to their lipid-lowering abilities [16, 17]. Based on the current literature, we aimed to investigate the effects of statins in an experimental model of OA by evaluating the percentage of MMP-3 expression in chondrocytes and OARSI OA cartilage histopathology scores.

## Marterials and methods

All procedures were in compliance with the Principles of Laboratory Animal Care (NIH publication No. 85–23, revised 1985) and Turkish Law 6343/2 Veterinary Medicine Deontology Regulation 6.7.26. The study was in compliance with Gazi University Ethical Council regulations, and the protocol was approved by Gazi University Experimental Animals Ethical Council on 27 December 2007 by decision code B.30.2.GÜN.0.EU.00.00/113-21239. The study was carried out in the Gazi University Experimental Animal Research Laboratory and was supported by Gazi University Department of Scientific Investigation Grant No: 01/2008-18.

### Animals and surgical procedure

Twenty-seven male albino Wistar rats weighing approximately 300 g each were obtained from Gazi University Experimental Animal Research Laboratory and were randomly divided into three groups of equal numbers. All surgical procedures were accomplished after intramuscular administration of 40 mg/kg ketamine hydrochloride (Ketalar, Pfizer Inc, USA) and 5 mg/kg xylazine hydrochloride. After being shaved and disinfected, right knees of animals in group 1 and group 2 underwent anterior cruciate ligament transection (ACLT) via a medial parapatellar arthrotomy to simulate knee instability. After surgery, the joint surface was washed with sterile saline solution, and both capsule and skin were sutured using Vicryl 4-0 (Ethicon, Edinburgh, UK) absorbable suture. Animals in group 1 were left untreated, whereas each animal in group 2 was treated with orally administered simvastatin (Merck Sharp & Dohme, Middlesex, UK) 20/mg per kilogram per day by gavage for 8 weeks starting from the first day of surgery. Animals in group 3 were sham operated with an arthrotomy only using the same approach. All animals were housed in different cages according to their groups, were allowed unlimited activity, and were sacrificed 8 weeks after the operation using an intravenous overdose of anesthesia. Twenty-seven knee joints in all groups were subsequently divided into femur (F) and tibia (T) subgroups. After disarticulation of the right knee joint, both femur and tibia were dissected from muscle, and cartilage was harvested from both medial and lateral tibial plateaus and femoral condyles.

### Tissue preparation, immunohistochemistry for MMP-3, and histopathology

Excisional biopsy specimens of proximal tibia and distal femur were fixed in 10% formalin for 1 week and then processed in 10% formic acid solution for decalcification. Formic acid solution was renewed in every 24 h, and the consistency of the bone was checked every day. The bone specimens could be cut at 72 h. Proximal tibia specimens were cut into two pieces in the sagittal plane, proximal femoral specimens were cut into two pieces in the coronal plane, and specimens were embedded in paraffin blocks to be sectioned on their cut surfaces. Three-micron-thick sections were cut on two slides: one to be stained by hematoxylin and eosin and the other by MMP-3 antibody. Immunohistochemical assays were performed on formalin-fixed, paraffin-embedded sections. After deparaffinization, sections were rehydrated with distilled water. Endogen peroxidase activity was blocked by 3% hydrogen peroxide for 10 min and the specimens were then washed in distilled water. Antigen retrieval was performed in citrate buffer pH 6.0 in a microwave oven at high temperature for 20 min for MMP-3. After standing for 20 min at room temperature, slides were washed in phosphate-buffered saline (PBS) pH 7.6 for 5 min. After protein blockage (Lab Vision Ultra V block, Fremont, CA, USA) for 10 min, tissue sections were incubated in primary antibodies against MMP-3 dilution 1/100 (Abcam, Cambridge, CB4, 0FW, UK) for 1 h at room temperature. After washing by PBS for 5 min, tissue sections were incubated in biotinylated secondary antibodies (Lab vision Fremont, CA, USA) for 20 min and then washed with PBS for 5 min. Slides were incubated in streptavidin/peroxidase complex (Lab vision Fremont, CA, USA) for 20 min and washed by PBS for 5 min. Sections were developed with the 3,3’ diaminobenzidine (DAB) as the chromogen and counterstained with hematoxylin. Sections were incubated overnight in primary antibodies against MMP-3 at 4°C. Placenta sections were used as positive controls for MMP-3.

The slides were screened by one pathologist. Positive staining was determined as cytoplasmic staining in chondrocytes. Total number of chondrocytes and positive-staining chondrocytes were counted in the femur and tibia, respectively, in three or more sections on 3–6 high-power fields (×400) according to the size of articular sections, and mean count of these sections was used to determine a proportion of cells expressing MMP-3. The pathologist was masked to the experimental procedures.

### Histopathological scores

Histopathological assessment was made using the OARSI cartilage OA histopathology assessment system by the same pathologist. This system is based on six grades and four stages, reflecting the severity and extent of OA over the joint surface. Grade is defined as OA depth progression into cartilage and is an index of severity or biologic progression of the osteoarthritic process. Grade is assessed by noting the most advanced grade present within cartilage, irrespective of its horizontal extent (grades 1–4: articular cartilage changes only; grades 5–6: cartilage and subchondral bone). Stage is defined as the horizontal extent of cartilage involvement irrespective of underlying grade. Score is defined as OA grade and OA stage (grade × stage). Therefore, scores represents a combined assessment of OA severity and extent [18, 19].

### Statistical analysis

Statistical comparisons were generated using Statistical Package for Social Sciences-11 for Windows program (SPSS, Chicago, IL, USA). All data are expressed as means ± standard deviation (SD). Histopathology and immunohistochemistry data were analyzed using a nonparametric Mann–Whitney *U* test. *P* values <0.05 were considered statistically significant.

## Results

**Fig. 1:**
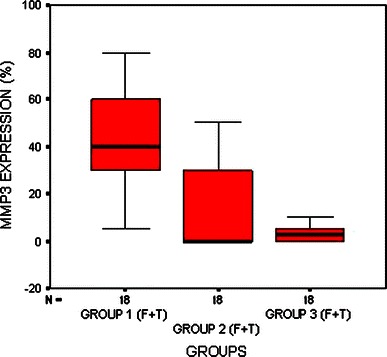
Distribution of chondrocyte matrix metalloproteinase-3 (MMP-3) staining of femur and tibia articular cartilage in three groups

**Fig. 2:**
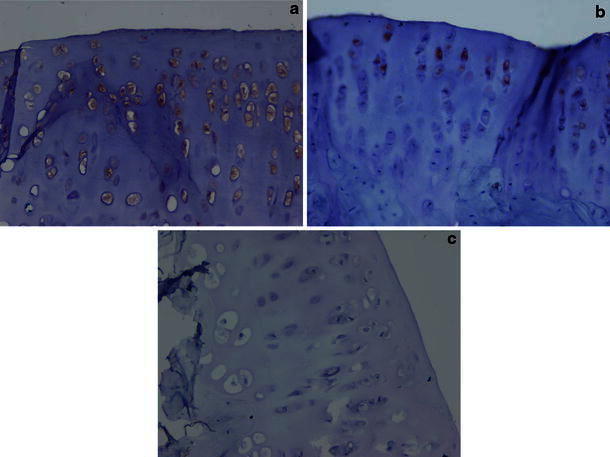
**a** Micrographs from group 1 (anterior cruciate ligament transection) distal femoral articular cartilage chondrocytes showing a high percentage of matrix metalloproteinase-3 (MMP-3) staining (MMP-3, immunoperoxidase ×20). **b** Micrographs from group 2 (anterior cruciate ligament transection + simvastatin) showing low percentage of MMP-3 staining (MMP-3, immunoperoxidase ×40). **c** Micrographs from group 3 (control knees) showing no MMP-3 staining (MMP-3, immunoperoxidase ×40)

Group 1 (ACLT) had a statistically significant high percentage of MMP-3 expression in chondrocytes compared with group 2 (ACLT + simvastatin), and simvastatin clearly suppressed MMP-3 expression in chondrocytes (*P* = 0.001; Fig. 1; Table 1). Group 3 (control) had lower MMP-3 staining compared with groups 1 and 2, whereas there was no statistically significant difference between the percentage of MMP-3 expression in chondrocytes between groups 2 and 3 (*P* = 0.429; Fig. 2a, b, c; Table 1).

**Table 1:** Articular cartilage matrix metalloproteinase-3 (MMP-3) expression and Osteoarthritis Research Society International (OARSI) histopathology scores in all groups

	Group 1	Group 2	Group 3
MMP-3 expression (%)	42.5 ± 21.0 (5–80)	14.4 ± 19.8 (0–50)	3.6 ± 4.1 (0–10)
	*P* = 0.001	*P* = 0.429	
OARSI histopathology scores	9.4 ± 3.9 (4–16)	5.1 ± 3.6 (1–9)	–
	*P* = 0.004	*P* < 0.001

**Fig. 3:**
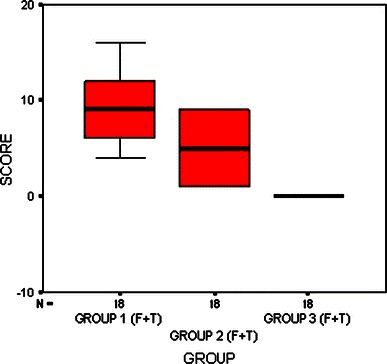
Distribution of Osteoarthritis Research Society International (OARSI) osteoarthritis (OA) pathology assessment scores of femur and tibia in all groups

Group 2 had a significantly higher OARSI cartilage OA histopathology assessment score than that of group 3 (*P* < 0.001), showing the ongoing osteoarthritic process, whereas scores in group 2 were lower compared with group 1, indicating the decrease in the extent and severity of OA (*P* = 0.004; Fig. 3; Table 1).

**Fig. 4:**
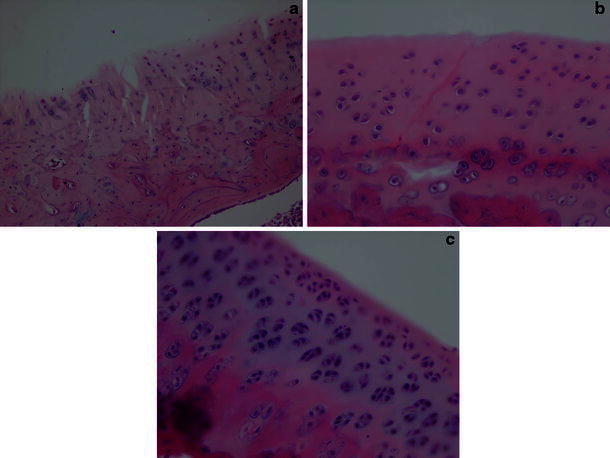
**a** Micrographs from group 1 (ACLT) showing matrix discontinuity at the superficial zone, matrix vertical and branched fissures into mid zone [hematoxylin and eosin (H&E) ×10]. **b** Micrographs from group 2 (ACLT + simvastatin) showing only superficial fibrillation (H&E ×20). **c** Micrographs from group 3 (control knees) showing a intact surface and normal matrix architecture (H&E ×20)

Despite group 1 (ACLT) having matrix discontinuity at the superficial zone, vertical and branched fissures into the mid zone, disorganization, and loss of collagen content, group 2 (ACLT + simvastatin) had only superficial fibrillation and did not show disorganization and loss of collagen content. Group 3 (control knees) had an intact surface and normal matrix architecture (Fig. 4a, b, c).

## Discussion

In this study, using a model of mechanically induced knee OA in rats, we demonstrated that simvastatin, a HMG-CoA reductase inhibitor, decreased the extent and severity of OA by reducing MMP-3 expression in articular cartilage. Most clinically prominent knee OA has a neglected history of trauma. Malalignment and instability, resulting in alteration of loading of the articular cartilage, initiates complicated biochemical pathways, leading to matrix degradation. In particular, repetitive shear and tangential traction forces on the surface of the cartilage activates TNF-α, IL-1, and MMP-mediated catabolic pathways. Therefore, orthopedic surgeons must be aware of the inflammatory phase and its outcomes, even after a minor trauma. In surgically induced models, radiographic, morphologic, and biochemical changes have been characterized in different animal models using dogs, rabbits, and rats [20–22]. We created joint instability by transecting the anterior cruciate ligament in a rat model and found that increasing mechanical stress and altering loading on the articular cartilage is characterized by progressive and gradual changes in the histology of articular cartilage. We believe that this model can mimic the pathogenesis of human OA in terms of cartilage degradation after similar traumas. This rat OA model with ACLT offered the advantages of relatively small size, low cost, and rapid progression of cartilage degeneration in studying and evaluating the effects of simvastatin on articular cartilage. Novel experimental studies have demonstrated that OA is not only a degenerative disease but also consists of an ongoing inflammatory process with anabolism and catabolism [1–5]. Regarding this important data, our mechanically induced OA model demonstrated cartilage degradation in a short period of time as a result of the ongoing inflammatory process.

During the inflammatory process although many proteinases belonging to all classes are expressed in joint tissues with OA, among them, MMPs are known to have a key role in joint destruction. According to these findings, we believe that blockade of this enzyme may be essential for preventing the disease. Statins, HMG-CoA (3-hydroxy-3-methylglutaryl-coenzyme A) reductase inhibitors, also have a proven role as an immunomodulatory and anti-inflammatory agent independent from their plasma-cholesterol-lowering effects [17]. These anti-inflammatory effects of statins were first demonstrated in coronary artery disease, in which statin treatment inhibited arterial-wall inflammation, stabilized atherosclerotic plaques, and reduced cardiovascular morbidity and mortality rates. Studies have confirmed that statins have a broad range of effects on cells and tissues involved in the inflammation process. Statins inhibit the production of IL-1b from human peripheral blood mononuclear cells, MMP 1-3-9 from rabbit macrophages, MMP-3 from IL-1b-stimulated human chondrocytes, and monocyte chemoattractant protein-1(MCP-1) from human peripheral monocytes, which prevents circulating monocytes from migrating to the arterial wall [23, 24].

Although statins have been reported to decrease the production of MMP-3 from IL-1b-stimulated chondrocytes and MMP-1,-3,-9 from macrophages, the mechanism of their inhibitory effects on MMP production in various cells is still controversial. Some studies suggest that statins modulate chondrocyte metabolism by reducing prenylation of key signaling molecules that control expression of collagen-degrading enzymes [25]. Among statin derivates, simvastatin had the advantage of lipophilic properties and the ability to passively diffuse into cells at a dose-dependent rate. The method of statin administration is also an important issue, as various methods such as intraperitoneal and intra-articular administration have been used to investigate its suppressing effect on inflammation in arthritis models [26]. Oral administration—a simple, effective, and more physiologic procedure—avoided the side effects of parenteral use and was favored in this study. Although the effective concentrations used were higher than human therapeutic values for hypercholesterolemia (1.5 mg/kg per day), no reverse effects of simvastatin on cell viability was observed.

In light of these findings, the main goal must be to suppress the ongoing inflammatory process by inhibiting the matrix-degrading enzyme activity of MMPs. We hypothesize that simvastatin may decrease cartilage degradation and AO progression by inhibiting MMP-3 expression in chondrocytes. Assessed by immunohistochemical methods, MMP-3 staining in chondrocytes were found to be significantly decreased. Parallel to this finding, using histopathological methods, OA extent and severity in the simvastatin-treated group decreased compared with in the untreated group. These results support our hypothesis. Depending on the previous findings, down-regulation of MMP-3 by simvastatin itself may be sufficient to decelerate the degradation process in OA.

To the best of our knowledge, this is the first study to investigate the effects of orally administered simvastatin on OA cartilage by evaluating chondrocyte MMP-3 expression and articular cartilage histopathologic OA assessment score in a mechanically induced OA model. Simvastatin may be used as a disease-modifying agent by suppressing the inflammatory phase and has a promising role in OA prevention for humans. Future clinical and prospective studies are needed to investigate the therapeutic importance of this agent by inhibiting MMP-3 expression in humans, especially in the early phase of posttraumatic knee OA.
